# Predictors of physical activity and sedentary behaviours among 11-16 year olds: Multilevel analysis of the 2013 Health Behaviour in School-aged Children (HBSC) study in Wales

**DOI:** 10.1186/s12889-016-3213-8

**Published:** 2016-07-15

**Authors:** Kelly Morgan, Britt Hallingberg, Hannah Littlecott, Simon Murphy, Adam Fletcher, Chris Roberts, Graham Moore

**Affiliations:** Centre for the Development and Evaluation of Complex Interventions for Public Health Improvement (DECIPHer), School of Social Sciences, Cardiff University, Cardiff, UK; Research and Evaluation Branch, Public Health Strategy Division, Public Health and Health Professions Department, Welsh Assembly Government, Cardiff, UK

**Keywords:** Physical activity, Sedentary behaviour, Active travel, School, Policy, Environment

## Abstract

**Background:**

The present study investigated associations between individual- and school-level predictors and young people’s self-reported physical activity (total activity and moderate-to-vigorous activity) and sedentary behaviours.

**Methods:**

Individual-level data provided by the 2013/14 cross-sectional survey ‘Health Behaviour in School-aged Children (HBSC) study in Wales’ were linked to school-level data within the ‘HBSC School Environment Questionnaire’. The final sample comprised 7,376 young people aged 11-16 years across 67 schools. Multilevel modelling was used to examine predictors of total physical activity, moderate-to-vigorous physical activity (MVPA) and sedentary behaviours (screen-based behaviours).

**Results:**

Taking more physical activity (less than 5 days vs. 5 or more days per week), engaging in higher levels of MVPA (less than 4 hours vs. 4 or more hours per week) and reporting 2 or less hours of sedentary time were predicted by several individual level variables. Active travel to school positively predicted high levels of physical activity, however, gender stratified models revealed active travel as a predictor amongst girls only (OR:1.25 (95 % CI:1.05 - 1.49)). No school-level factors were shown to predict physical activity levels, however, a lower school socio-economic status was associated with a higher level of MVPA (OR:1.02 (95 % CI:1.01 - 1.03)) and a lower risk of sedentary behaviour (OR:0.97 (95 % CI:0.96 – 0.99)). A shorter lunch break (OR:1.33 (95 % CI:1.11 - 1.49)) and greater provision of facilities (OR:1.02 (95 % CI:1.00 - 1.05)) were associated with increased sedentary activity. Gender stratified models revealed that PE lesson duration (OR:1.18 (95 % CI:1.01 - 1.37)) and the provision of sports facilities (OR:1.03 (95 % CI:1.00 - 1.06)) were predictors of boy’s sedentary behaviours only.

**Conclusion:**

Shorter lunch breaks were associated with increased sedentary time. Therefore, while further research is needed to better understand the causal nature of this association, extending lunch breaks could have a positive impact on sedentary behaviour through the provision of more time for physical activity. The findings also suggest that active travel could offer a mechanism for increasing physical activity levels particularly amongst girls. Particularly, the design and evaluation of interventions to promote physical activity during school hours should employ a comprehensive approach, including a focus on school policies and behaviours both in and out of school hours.

## Background

Regular physical activity aids normal growth and development throughout childhood [1–3] and reducing a range of chronic disease risks [4], and is associated with improved mental health and wellbeing [5]. Current public health recommendations suggest that young people aged 5-17 years perform at least 60 minutes moderate to vigorous physical activity (MVPA) daily [6]. However, a large proportion of young people do not meet this recommendation, particularly girls; recent evidence from Wales shows that 11 % of girls compared to 20 % of boys are sufficiently active [7]. In addition, an emerging evidence base indicates that sedentary behaviour increases metabolic risk profiles, independent of physical activity [8]. International HBSC (Health Behaviour in School-aged Children) study findings from Scotland have shown that over 60 % of 15-year old girls and boys report two or more hours of television viewing per day [9], a threshold shown to reduce physical and psychosocial health outcomes amongst children and youth [10]. Hence promoting young people’s physical activity, while reducing sedentary behaviour, are dual priorities.

In attempting to improve adolescent health behaviours, school-based interventions are important for a number of reasons. The years young people spend at school are a formative period in their ‘health career’ [11] and health-risk behaviours such as sedentary behaviour become entrenched during this period [12, 13]. Where education is provided universally, schools provide access to the vast majority of young people and thus have potential to improve health at a population level [14]. Furthermore, the school environment itself can influence young people’s health in various positive and negative ways [15]. One recent Cochrane review concludes that school-based interventions can have short-term effects on young people’s physical activity levels [16], while another shows that multi-level interventions combining education, environmental change and family involvement have been effective across a range of behavioural domains including physical activity [17]. This is consistent with Ottawa Charter principles which argued that health promotion should move away from individualistic interventions and instead focus on how settings, such as schools, can support good health [18, 19].

UK studies suggest that a majority of young people’s overall physical activity takes place during the school day [20], with key opportunities during physical education (PE) and lunch breaks. Currently the UK government recommends that schools provide at least two hours of PE and sport a week, although there is no statutory minimum amount of time in England and Wales [21]. Previously, national estimates of PE provision across schools in England were measured using survey data on pupils aged 5-16 years, in 98 % of all state schools. Data from the Physical Education and Sport survey, conducted annually between 2003/4-2009/2010, show that 90 % of schools provided a minimum of two hours PE for pupils in year 1-7. However, from Year 8, schools reported less PE provision; and by years 10 and 11 this fell to 59 % and 56 % of schools respectively [22]. Although evidence suggests a positive relationship between physical activity and academic performance [23], PE time declines as academic pressures increase, around GCSE years. Recent analyses of these data showed that schools with more ethnic minority students and schools in more deprived areas offered less PE time while schools with a higher proportion of pupils receiving free school meals offered more [22].

In addition to PE, school lunch breaks provide an opportunity for young people to be physically active. However, recent decades have seen a trend toward shortening of school breaks in response to academic pressures and behavioural problems [24], potentially reducing opportunities for physical activity and increasing the proportion of time in seated sedentary activities. In a sample of Australian adolescents, Ridgers et al. [25] found that pupil’s physical activity levels decreased and sedentary levels increased during lunch breaks and recess over time; similar to overall daily physical activity levels. Nevertheless, lunch breaks and recess contributed 10.7 % and 6.6 % respectively, towards moderate- and vigorous daily physical activity. It is plausible that restricting the length of time available for play will limit physical activity, while encouraging young people to spend more of the school day in seated sedentary behaviour will encourage sedentary behaviour to be carried forward into leisure time. To date, no studies have examined links between the duration of school lunch-breaks and adolescent’s physical activity, or sedentary behaviour. Among young children, active play at recess is initially intense and then markedly decreases [26], which may contribute to observed negative associations between increased recess duration and physical activity due to children becoming fatigued and bored more quickly [27]. However, adolescents may have more stamina and be able to focus on activities for longer durations.

In addition to the school day, Van Sluijs et al. [28] report that active travel to and from school can contribute up to half of a young person’s overall physical activity. A number of previous studies [29, 30] and systematic reviews [31, 32] also demonstrate that a significant volume of young people’s overall physical activity can be attributed to active school transport. Fewer studies have examined associations between active travel to school and sedentary behaviours, with no clear evidence in either direction to date [31]. Hence, whilst promotion of active travel to school remains a popular mechanism for promoting young people’s physical activity, less is known about its potential to influence young people’s sedentary behaviours outside of school.

Examining differences in associations by gender is also important. Compared to girls, boys are generally more physically active during the day [33] and during lunch breaks [27], but spend more time in screen-based behaviours [34]. They are also more likely to actively commute to school [35] and associations between active travel and physical activity may be stronger for boys than for girls [36]. In school, boys are also provided significantly more minutes of PE on average per week compared to girls [22]. Understanding how physical activity levels and sedentary behaviour are differently associated with school and individual characteristics can help identify strategies for more effective interventions for boys and girls respectively.

This paper reports analyses of cross-sectional data from the November 2013 to March 2014 HBSC survey. The aim of this study was to examine associations between individual- and school-level predictors and self-reported physical activity (total activity and moderate-to-vigorous activity) and sedentary behaviours in young people.

## Methods

The data used in this study were collected as part of the November 2013 to March 2014 cross-sectional survey ‘Health Behaviour in School-aged Children (HBSC) study in Wales’. Completed by 9,055 secondary school students aged 11 to 16-years, the questionnaire provides a nationally representative sample from 82 secondary schools across Wales. The survey is part of a wider World Health Organisation (WHO) Cross-National survey involving 44 countries. Data were gathered from schools between November 2013 and March 2014, with secondary schools chosen through using a two-stage sampling process: First, schools were stratified according to local authorities and free-school meal eligibility and randomly selected; and second, schools were asked to randomly select one class (approximately 25 students) in each of the year groups 7 (aged 11-12 yr), 8 (12-13 yr), 9 (13-14 yr), 10(14-15 yr) and 11(15-16 yr) for participation within the survey. Participants were assured of anonymity and confidentiality and data collection took place within the classroom environment under exam conditions. Further details on the HBSC study procedures can be accessed elsewhere [37, 38].

This study also uses data provided by a member of the senior management team (60 % of data provided by a Deputy- or Assistant Headteacher) within a secondary school who completed the HBSC 2013/2014 School Environment Questionnaire (n = 7,376). Head teachers from each school participating in the HBSC study were invited to participate and schools received £150 to cover any costs incurred through participating. Of the 82 participating schools, 67 completed a school environment survey. Resultantly, the total sample analysed in this paper consisted of the 7,376 students within these 67 schools, with both individual- and school-level data available.

### Measures of physical activity and sedentary behaviours

The HBSC survey asks two questions regarding *physical activity levels*. First, students are asked “over the past 7 days, on how many days were you physically active for a total of at least 60 minutes per day” (responses: “every day”, “4 to 6 days a week”, “2 to 3 times a week”, “once a week”, “once a month”, “less than once a month”, “never”); students reporting 5 or more days per week were classed as physically active. This threshold, as opposed to 7 days a week, was chosen as only 15.6 % of the sample reported being physically active every day. Second, *moderate to vigorous physical activity* (MVPA) was assessed by asking students, “outside of school hours, how many hours in a week do you usually exercise in your free time so much that you get out of breath or sweat?” (responses: “none”, “half an hour”, “about 1 hour”, “about 2 to 3 hours”, “about 4 to 6 hours”, “about 7 hours or more”). Participants reporting 4 or more hours per week were classed as achieving sufficient MVPA.

Three questions regarding screen-based sedentary patterns during spare time (hours per day on weekdays) were asked. They concerned; 1) time spent watching TV, videos and entertainment on a screen, 2) time spent playing games on a computer, games console or tablet/smart phone, and 3) time spent using electronic devices such as computers, tablets or smart phones for other purposes. The response options for each question took the same format (“none at all”, “about half an hour a day”, “one hour a day”, “two hours a day”, “three hours a day”, “four hours a day”, “five hours a day”, “6 hours a day” and “about 7 or more hours a day”). Students reporting more than two hours per day for any of the three questions were regarded as sedentary. Response categories concerning two hours or less were coded 0 and responses of three hours or more were coded 1. Within the discussion section, these groups are referred to as low and high sedentary behaviours, respectively.

### Individual-level variables

Participants reported their *age* (year and month of birth), *gender* (boy/girl)*,* and *ethnicity* (responses: “White”, “Mixed Race”, “Asian or Asian British”, “Black or Black British”, “Chinese” and “Other”; those reporting White were catergorised as 0 and all other reponses coded as 1 (referred to as Black Minority Ethnic (BME))). Participants were asked a two-part question concerning both smoking and alcohol consumption: ‘In your lifetime and in the last 30 days; “On how many days (if any) have you smoked?” and “On how many days (if any) have you drunk alcohol?” (Responses: “Never”, “1-2 days”, “3-5 days”, “6-9 days”, “10-19 days”, “20-29 days”, and “30 days or more”). BMI (Body Mass Index) was calculated from a participant’s report of height and weight measures.

#### Active travel

Participants were asked to choose one response to the following question; “On a typical day is the main part of your journey to school made by walking, bicycle, bus/train/tram, car/motorcycle or other means?” Responses “walking” and “bicycle” were coded 1 signifying active travel and all other responses coded 0.

#### Family Affluence Score (FAS)

FAS [39, 40], an indicator of child material affluence, was derived from 6 survey items which asked; 1) “Do you have your own bedroom?” 2) “How many computers does your family own?” 3) “Does your family own a car, van or truck?” 4) “Does your family have a dishwasher at home?” 5) “How many bathrooms are in your home?” and 6) “How many times did you and your family travel out of Wales on holiday/vacation last year?” The scores for each item were summed to give a total affluence score.

### School-level variables

#### Free-school meal entitlement (FSM)

Providing a measure of school-level socioeconomic status, the percentage of FSM entitlement within each school was identified. Free school meals are offered in Wales to school students whose parents are in receipt of a range of state benefits such as Child Tax Credit. FSM scores were divided into three tertiles; low (<10 % entitled to FSM), medium (11-19 % eligible) and high (>19 % entitled).

#### Duration of lunch break

Head teachers were asked “How long do children have for their lunch break at your school?” (responses: “Less than 20 minutes”, “30-35 minutes”, “40-45 minutes”, “50-55 minutes”, and “60 or more minutes”). Responses indicating less than 50 minutes were coded 0 and responses of 50 minutes or more coded 1.

#### Physical education duration

For each year group (years 7-11) headteachers stated the number of minutes of PE timetabled weekly within the formal curriculum. Responses were later coded into the following categories; less than 60 minutes, 60 to 89 minutes, 90 to 119 minutes and 120 minutes or more.

#### Existing school health policy

Headteachers were asked “Does your school currently have a specific healthy eating or Food and Fitness policy?” Possible responses were; “Yes, written policy in place”, “currently developing a written policy” and “No”; those who reported yes to an existing policy were coded 1 and both alternative responses a 0.

#### Provision of sports facilities

Head teachers were asked to state which sports facilities (10 items listed: gymnasium/sports hall, dance/fitness studio, swimming pool, running track, sports field/grass pitches, basketball/netball courts, 5-a-side football pitches, playground, skateboard area and equipment for team sports.) were available for pupils on site and to specify the time when each facility was available (as part of PE lessons, during lunch and after school). Each opportunity for students to use sports facilities throughout the day was counted and summed for each school (i.e. the number of facilities available was multiplied by the number of times students could use them to provide an overall score).

### Statistical analyses

All analyses were undertaken in Stata (V.14.0). To account for the nature of the HBSC survey data, typically nested within a hierarchical structure, we employed multilevel models with a two-level structure. Level 1 was represented by school students and level 2 represented by schools. Individual models were run to represent each of the following study outcomes; (1) physical activity (5 days a week vs. less than 5 days), (2) MVPA (4 or more hours a week vs. less than 4 hours) and (3) sedentary behaviours (more than 2 hours per day vs. 2 hours or less a day).

Three models were constructed for each study outcome. First, a null model was developed, including school as a random effect, given the statistically significant (*p* < 0.05) likelihood ratio (LR) test statistic. Second, individual-level variables including; age, gender, ethnicity, FAS and active transport were entered as fixed effects into the model. Random coefficients and plausible two-way interactions were also entered into the model and retained if they revealed an improved model fit (judged by LR test statistic). Third, school-level predictors (i.e. FSM, lunch time duration, PE lesson duration, policy and provision of facilities) were entered into the model. The Intracluster Correlation (ICC) is provided for each model, denoting the amount of dependency among observations within a group. For physical activity outcomes, although we defined thresholds lower than public health recommendations (i.e. that children should participate in 60 minutes of activity every day) to maximise statistical power, as a sensitivity analysis we reran analyses using more stringent thresholds. Odds ratios in these sensitivity analyses were similar, though with wider confidence intervals due to more limited power. Hence, we report only the models using cut-points described above.

## Results

School- and individual level questionnaires were available for 7,376 young people (49.1 % girls). Descriptive statistics can be seen in Table 1 and information concerning each predictor variable entered into the analyses in Table 2. BMI data were available for 39.3 % of young people, a response rate similar to other countries reporting HBSC data [41]. BMI, smoking and alcohol consumption data were used for descriptive information only and are therefore not included within analyses. Of the total sample, 40 % of students reported being physically active for 5 or more days a week and 28 % reported 4 or more hours of MVPA a week. In comparison to girls, boys reported more physical activity (mean days/week; 4.31 vs. 3.65, *p* < 0.001) and MVPA (mean hours/week; 2.95 vs. 2.27, *p* < 0.001), though also reported more sedentary behaviour (mean hours/day; 7.86 vs. 7.04, *p* < 0.001). Boys were also more likely to actively travel to school (31.0 % vs. 28.9 %, *p* = 0.049). On average, participants reported 7.5 hours (SD 4.7) of total screen-based behaviours on weekdays (accounting for time spent watching TV, playing computer games and using computers for non-gaming purposes). Results from initial analyses revealed significant school-level variance in all study outcomes prior to individual-level predictors being added to the analyses, with intra-cluster correlations substantially stronger for sedentary behaviour than for physical activity.

**Table 1:** Descriptive data for the sample

Characteristic	N*	Mean(SD)	%(n)
Individual level			
Age (yr)	7345	13.7 (1.4)^	
Female	7350		49.1 (3607)
White	7342		92.7 (6808)
Overweight or obese	2899		18.2 (527)
Physically active (days/week)	7279	4.0 (2.0)	
MVPA (hrs/week)	6943	2.6 (2.3)	
Actively travelling to school	7123		29.0 (2135)
Actively travelling from school	7059		36.3 (2560)
Screen-based behaviour > 2 hours per weekday	7376		84.9 (6259)
Typical alcohol consumption ≥ 3 drinks	7292		15.2 (1110)
Smoke at least once a week	7358		3.2 (237)
Family Affluence Score	7200	15.0 (2.3)	
School level			
Free School Meal eligibility	7376	15.9 (8.7)	
Lunch break duration ≥ 50 minutes	7267		58.5 (4254)
Existing food and fitness policy	7302		73.0 (5329)
Extra-curricular sports activities delivered by PE staff	7376		97.8 (7210)
PE curriculum time allocated each week ≥ 60 minutes	7250		94.6 (6855)
Number of sports facilities available after school	7376	5.5 (2.2)	
Number of sports facilities available during lunch	7376	5.2 (2.3)	

* Number completing the survey item

^ 4 young people were outside of the 11-16 age range

**Table 2:** Information concerning each predictor included within the analyses

Variable	Description	Range	Mean (SD)
Individual level			
Age	Age at completion of questionnaire	10.9- 17.8	13.7 (1.4)
Gender	Boy (0)/Girl (1)		N/A
Ethnicity	White (0)/BME (1)		N/A
Family Affluence Score (FAS)	Derived from six constructs	7-19	15.0 (2.3)
Active travel	Other mode(0)/Actively (walk/bike) travels to school daily(1)	Yes/No	N/A
School level			
Free school meal eligibility (FSM)	% of students receiving free meals at each school	0-39.5	15.9(8.7)
Duration of lunch break	Categorical (minutes);		N/A
Time allocated to PE lessons	Categorical variable (minutes); up to 60 (1), 60-89 (2), 90-119 (3) and 120 or more (4)	1-4	N/A
Existing food and fitness policy	No (0)/Yes (1)		N/A
Number of sports facilities available	Opportunities during/after school to use sports facilities	3-27	17.2 (5.5)

Table 3 presents odds ratios and 95 % confidence intervals from multilevel logistic regression models for each study outcome. Findings show that taking more total physical activity, engaging in higher levels of MVPA and reporting 2 or less hours of sedentary time were predicted by a range of individual level variables. A higher age was associated with less total physical activity and more sedentary behaviour, though also with a higher level of MVPA. White ethnicity was associated with higher levels of MVPA (though not related to total physical activity or sedentary behaviour). A higher level of family affluence was associated with a higher level of total physical activity and MVPA, though not with sedentary behaviour. Children who actively travelled to school reported higher levels of total physical activity, though not MVPA. However, a trend toward increased sedentary behaviour among active commuters approached significance (*p* = 0.053). Of the school-level variables included within the model, there were no significant correlates of total physical activity. A lower FSM entitlement was associated with a higher level of MVPA and a lower risk of sedentary behaviour. Sedentary behaviour was also associated with lunch break length and the provision of school facilities, with a shorter lunch break and greater provision of facilities associated with increased sedentary activity. Within unadjusted models, school level intracluster correlations (ICC) ranged from 0.02 (total physical activity) to 0.06 (sedentary behaviour). Models inclusive of individual level predictors revealed mostly declining ICCs. In final models, adjusting for both individual- and school-level predictors, ICCs for total physical activity and MVPA remained constant whilst the ICC for sedentary behaviour declined from 0.06 to 0.03. In separate models for boys and girls (Table 4), most correlates are similar. However, active travel is more strongly associated with physical activity for girls than for boys. For boys, there is a significant association between sedentary behaviour and two school level variables; time allocated to PE lessons and the provision of sports facilities.

**Table 3:** Odds ratios and 95 % confidence intervals from multilevel logistic regression analysis of correlates of physical activity, MVPA and sedentary behaviour

		Physical activity	MVPA	Sedentary behaviour
		(n = 6,499)	(n = 6,216)	(n = 6,576)
Individual level	Age	**0.88 (0.84 - 0.91)**	**1.11 (1.06 - 1.16)**	**1.40 (1.33 – 1.47)**
	Sex*	**0.53 (0.48 - 0.59)**	**0.55 (0.49 - 0.62)**	**0.61 (0.53 – 0.70)**
	Ethnicity^	0.98 (0.79 - 1.21)	**0.76 (0.58 - 0.99)**	0.91 (0.69 - 1.21)
	FAS	**1.12 (1.10 - 1.15)**	**1.16 (1.13 - 1.19)**	0.98 (0.95 - 1.01)
	Active travel~	**1.18 (1.05-1.33)**	1.02 (0.89 - 1.17)	1.18 (1.00 - 1.39)
School level	FSM	**0.99 (0.98 - 1.00)**	**0.98 (0.97 – 1.00)**	**1.03 (1.01 – 1.04)**
	Lunch break duration	0.91 (0.75 - 1.10)	1.06 (0.88 - 1.28)	**0.67 (0.51 – 0.89)**
	PE time	1.00 (0.92 - 1.10)	1.03 (0.95 - 1.12)	1.06 (0.94 - 1.19)
	Policy	0.98 (0.83 - 1.15)	1.05 (0.89 - 1.23)	1.01 (0.80 - 1.28)
	Facilities	1.01 (1.00 - 1.03)	1.00 (0.99 - 1.02)	**1.02 (1.00 - 1.05)**
ICC – constant only		0.02	0.03	0.06
ICC – level 1 variables		0.01	0.01	0.06
ICC – Level 1 & 2 variables		0.01	0.01	0.03

*Girls are the reference group ^ethnic minority is reference group ~ active travellers are the reference group

FAS, Family Affluence Score; FSM, Free school meal eligibility; PE time, Physical Education time, ICC, Intraclass Correlation Coefficient. Bold values signify significant findings *P* < 0.05

**Table 4:** PA, MVPA and less sedentary behaviour by gender (Boys n = 3,453, Girls n = 3,320)

		Physical activity		MVPA		Sedentary behaviour	
		Boys	Girls	Boys	Girls	Boys	Girls
		(n = 3,342)	(n = 3,157)	(n = 3,176)	(n = 3,040)	(n = 3,388)	(n = 3,188)
Individual level variables	Age	**0.92** **(0.88 - 0.97)**	**0.82** **(0.78 - 0.87)**	**1.12** **(1.06 - 1.18)**	**1.09** **(1.02 - 1.16)**	**1.32** **(1.22 – 1.43)**	**1.46** **(1.36 – 1.56)**
	Active travel	1.13 (0.97 - 1.32)	**1.25** **(1.05 - 1.49)**	1.05 (0.88 - 1.25)	0.97 (0.77 - 1.21)	1.17 (0.91 - 1.50)	1.21 (0.97 – 1.51)
	Ethnicity	1.05 (0.85 - 1.38)	0.92 (0.66 - 1.28)	0.83 (0.60 - 1.16)	0.64 (0.40 – 1.02)	0.84 (0.58 - 1.27)	1.00 (0.68 - 1.48)
	FAS	**1.11** **(1.08 - 1.15)**	**1.13** **(1.10 - 1.18)**	**1.13** **(1.09 - 1.17)**	**1.20** **(1.14 - 1.25)**	0.99 (0.95 - 1.04)	0.97 (0.93 - 1.01)
School level variables	FSM	0.99 (0.98 - 1.00)	0.99 (0.98 - 1.01)	**0.99** **(0.98 - 1.00)**	**0.98** **(0.96 - 1.00)**	**1.04** **(1.02 – 1.06)**	**1.02** **(1.00 – 1.04)**
	Lunch break duration	0.98 (0.82 - 1.18)	0.86 (0.65 - 1.13)	1.01 (0.83 - 1.24)	1.17 (0.83 - 1.63)	**0.67** **(0.47 – 0.96)**	**0.65** **(0.48 – 0.88)**
	PE time	0.9 (0.88 - 1.04)	1.04 (0.92 - 1.17)	1.00 (0.91 - 1.10)	1.07 (0.93 - 1.23)	**1.18** **(1.01 - 1.37)**	0.99 (0.87 - 1.12)
	Policy	0.88 (0.75 – 1.03)	1.07 (0.84 - 1.38)	1.00 (0.83 - 1.19)	1.16 (0.87 - 1.56)	1.33 (0.99- 1.79)	0.82 (0.63 - 1.07)
	Facilites	1.01 (0.99 - 1.02)	1.02 (0.99 - 1.04)	0.99 (0.98 - 1.01)	1.01 (0.98 - 1.03)	**1.03** **(1.00 - 1.06)**	1.02 (1.00 - 1.04)
ICC – level 1 variables		0.02	0.02	0.04	0.04	0.04	0.04
ICC – Level 1 & 2 variables		0.02	0.02	0.02	0.02	0.01	0.01

*FAS* Family Affluence Score, *FSM* Free school meal eligibility, *PE time* Physical Education time, *ICC* Intraclass Correlation Coefficient. Bold values signify significant findings *P* < 0.05

## Discussion

In the present study, most young people reported being active 4 or less days per week, with 16 % meeting current physical activity recommendations. Consistent with previous reports which have used self-reported and objective measures [42–44], boys were almost twice as likely to be physically active (for 5 or more hours per week) and engage in sufficient levels of moderate-vigorous physical activity (based on the study criteria of ‘4 or more hours per week after school’) in comparison to girls. Furthermore, a considerable proportion of our study sample (85 %) exceeded the recommended levels of 2 or less hours of screen-based activities per day [10]. With an average of 7.5 hours screen-based activities per day, our observations are largely consistent with objectively measured sedentary behaviours of US and Canadian adolescents [45]. Furthermore, despite reporting higher levels of physical activity and more MVPA, we also found that boys reported higher levels of sedentary behaviour in comparison to girls. A number of studies [46–49] have observed comparable findings, including te Velde et al., [50] who studied a cross-sectional sample of 9-14 year olds in nine European countries. The notion that high levels of physical activity and high sedentary behaviours can co-exist, infers an asymmetrical association between the two behaviours [51]. Adopting a cluster analysis approach, Jago et al., [47] also identified adolescents who reported a ‘high activity high sedentary’ lifestyle. Dissecting the hours during which activity patterns occurred, they found that this particular group accrued most of their physical activity in school hours and the majority of sedentary time after school [47]. Our finding that boys were more sedentary in comparison to girls could reflect the type of sedentary measure used (i.e. ‘screen-based time’). It is possible that if measures such as ‘time spent listening to music’ or ‘time spent talking on the phone’ were collected, we would observe higher levels of sedentary behaviours in girls [10].

Active travel was positively associated with physical activity levels in girls; those who actively travelled to school had a 25 % increased chance of being sufficiently physically active in comparison to girls who did not actively travel. This is consistent with other studies which have reported increasing physically activity levels amongst active travellers [32, 52], although there is conflicting data regarding whether effects are strongest for girls [53], or boys [29]. Our findings suggest that active travel might be an important contributor to daily physical activity levels for girls, although boys are generally more active through other means. Notably, before gender-stratified analyses, our findings also revealed a trend towards increased sedentary behaviours amongst active commuters. Few studies have examined associations of active travel with sedentary behaviour previously and findings are to date inconsistent [54]. However, the findings of the present study tentatively indicate that interventions focused on promoting active travel for example should perhaps also include components which aim to discourage sedentary behaviour.

School level predictors including lunch-break length, duration of PE time, PE facilities and school food and fitness policies were however not associated with physical activity. However, we observed significant associations between some school-level predictors and sedentary behaviours for both boys and girls. First, a shorter lunch break was shown to be associated with higher sedentary behaviours among both genders. Given that the measures of sedentary behaviour focused primarily around leisure time activities (i.e. television viewing and game-playing) it is perhaps plausible that where young people are encouraged to spend much of their day seated through limiting the duration of time available for movement, this tendency for sedentary behaviour carries forward into leisure time. Previous reports have shown large variation between school lunch break durations in Wales, with some lunch breaks less than 30 minutes [55]. There is a paucity of literature surrounding lunch break duration in secondary schools and associations with young people’s physical activity and sedentary behaviours. Secondary schools providing less than 45 minute lunch breaks have however, been linked to poorer food choices and lower nutrient intakes in children [55, 56]. Further research examining how exactly young people spend their time during lunch break is required with use of objective activity measures and direct observations [57, 58]. This would provide a greater understanding of the social influences and school-setting on young people’s physical activity levels.

Second, though not significant in whole group analysis or for girls, among boys, longer PE lessons and a higher provision of school facilities were associated with high sedentary behaviours. These findings, accompanied by the lack of evidence of increased overall physical activity associated with increased PE time, could indicate a compensatory mechanism [59], whereby young people who are exposed to more physical activity throughout the school-day will compensate with sedentary behaviours after school hours. Previous findings from a recent longitudinal study [60] have shown that levels of physical activity during- and after-school hours become increasingly displaced with sedentary behaviours throughout ages 12 to 15 years. Furthermore, with ever evolving technologies and reports of children ‘preferring to watch television or play computer games as opposed to being physically active’ [61], it is important to be concerned with high levels of sedentary behaviours even amongst those who are provided with physical activity opportunities in the school day.

### Limitations

This paper has both strengths and limitations. Notably, our study utilises a large scale, nationally representative survey of 11-16 year-olds attending secondary schools throughout Wales. Further analysis of HBSC datasets from other countries would provide regional comparisons between activity levels within different school-settings and different policy settings. The main limitation of this study is the reliance on self-reported data, with both individual- and school-level factors dependent upon the reports and interpretations of young people and members of the school senior management team. Validation studies have reported an overestimation of activity when using questionnaires to gather physical activity measures amongst adolescents [62]. Despite providing detailed and objective measures of physical activity however, the use of devices such as accelerometers would not be practical with such a vast sample size and may introduce response bias [63]. Furthermore, it would have been desirable to have physical activity and sedentary questions which were directed towards specific periods throughout the day i.e. during lunch breaks, during PE lessons, after school etc. This would clarify that ‘free-time’ is defined as time outside of school hours. Future research will look to separate physical activity levels and sedentary behaviours within these particular time contexts. It would have also been advantageous to include BMI data within our analyses, given the potential interactions with our study outcomes; however, due to the incompleteness of data this was not feasible. As with all cross-sectional studies, we are unable to confirm the causal pathways underlying the associations observed within our study findings.

## Conclusions

Active travel to school could offer a mechanism for increasing physical activity levels, particularly amongst girls as part of a whole-school approach. Our findings also highlight the association between shorter lunch breaks and increased sedentary time. Based on our findings and studies addressing dietary impacts [55], there is growing evidence that if schools maintain or extend the duration of lunch breaks, this may have a positive impact on sedentary behaviour through the provision of more time for physical activity. Hence, the design and evaluation of interventions to promote physical activity during school hours should employ a comprehensive approach encompassing school policies and measures of behaviour both in- and out of school hours.

## Abbreviations

MVPA, Moderate to Vigorous Physical Activity; HBSC, Health Behaviour in School-aged Children; PE, Physical Education; WHO, World Health Organisation; BMI, Body Mass Index; BME, Black Minority Ethnic; FAS, Family Affluence Score; FSM, Free-School Meal entitlement; LR, Likelihood Ratio; ICC, Intracluster Correlation; OR, Odds Ratio; CI, Confidence Interval
